# Hemodynamic responses to handgrip and metaboreflex activation are exaggerated in individuals with metabolic syndrome independent of resting blood pressure, waist circumference, and fasting blood glucose

**DOI:** 10.3389/fphys.2023.1212775

**Published:** 2023-08-07

**Authors:** Jon Stavres, Ryan A. Aultman, Caleb F. Brandner, Ta’Quoris A. Newsome, Anabelle Vallecillo-Bustos, Havens L. Wise, Alex Henderson, Diavion Stanfield, Joseph Mannozzi, Austin J. Graybeal

**Affiliations:** ^1^ School of Kinesiology and Nutrition, University of Southern Mississippi, Hattiesburg, MS, United States; ^2^ Department of Physiology, Wayne State University School of Medicine, Detroit, MI, United States

**Keywords:** exercise pressor response, PECO, metabolic disease, blood pressure, cardiovascular

## Abstract

**Introduction:** Prior studies report conflicting evidence regarding exercise pressor and metaboreflex responses in individuals with metabolic syndrome (MetS).

**Purpose:** To test the hypotheses that 1) exercise pressor and metaboreflex responses are exaggerated in MetS and 2) these differences may be explained by elevated resting blood pressure.

**Methods:** Blood pressure and heart rate (HR) were evaluated in 26 participants (13 MetS) during 2 min of handgrip exercise followed by 3 min of post-exercise circulatory occlusion (PECO). Systolic (SBP), diastolic (DBP), and mean arterial pressure (MAP), along with HR and a cumulative blood pressure index (BPI), were compared between groups using independent samples *t*-tests, and analyses of covariance were used to adjust for differences in resting blood pressure, fasting blood glucose (FBG), and waist circumference (WC).

**Results:** ΔSBP (∼78% and ∼54%), ΔMAP (∼67% and ∼55%), and BPI (∼16% and ∼20%) responses were significantly exaggerated in individuals with MetS during handgrip and PECO, respectively (all *p* ≤ 0.04). ΔDBP, ΔMAP, and BPI responses during handgrip remained significantly different between groups after independently covarying for resting blood pressure (*p* < 0.01), and after simultaneously covarying for resting blood pressure, FBG, and WC (*p* ≤ 0.03). Likewise, peak SBP, DBP, MAP, and BPI responses during PECO remained significantly different between groups after adjusting for resting blood pressure (*p* ≤ 0.03), with peak SBP, MAP, and BPI response remaining different between groups after adjusting for all three covariates simultaneously (*p* ≤ 0.04).

**Conclusion:** These data suggest that exercise pressor and metaboreflex responses are significantly exaggerated in MetS independent of differences in resting blood pressure, FBG, or WC.

## Introduction

Metabolic syndrome (MetS) is a clinical condition characterized by the accumulation of specific cardiometabolic risk factors, including abdominal obesity, insulin resistance, hypertension, and dyslipidemia (Grundy et al., 2005). According to the most recently available data, approximately 33% of U.S. adults suffer from MetS (Aguilar et al., 2015), the prevalence of which appears to be growing fastest in young adults (Hirode and Wong, 2020). Considering the presentation of MetS (and its components) is associated with a significant increase in the risk of developing cardiovascular or metabolic disease (Isomaa et al., 2001; Laaksonen et al., 2002; Hu et al., 2004; Li et al., 2016; Lee et al., 2020), this condition presents a unique challenge to the U.S. healthcare system. Accordingly, it is very important for researchers and clinicians to understand the pathophysiology of MetS, as this will ultimately inform the development of effective mitigation strategies.

One aspect of MetS that is of particular interest to clinicians is the manifestation of cardiovascular dysfunction. Resting autonomic dysfunction is a hallmark characteristic of both cardiovascular and metabolic disease and is expressed as reductions in heart rate variability [HRV; (Falcone et al., 2014; Utriainen et al., 2018)], elevated resting sympathetic tone (Grassi et al., 2003; Barretto et al., 2009), and impaired baroreflex control (Creager and Creager, 1994; Grassi et al., 2009; Bakkar et al., 2020). Exaggerated exercise pressor responses, which are governed by a combination of feed-forward [i.e., central command (Goodwin et al., 1972; Eldridge et al., 1981; Gandevia et al., 1993)] and feed-back [i.e., group III and IV muscle afferents (Alam and Smirk, 1937; Kaufman et al., 1983; Amann et al., 2010)] neural pathways, are also prevalent in many cardiovascular and metabolic diseases. Examples include hypertension (Delaney et al., 2010; Greaney et al., 2014), peripheral artery disease (Muller et al., 2012; Luck et al., 2017), and type 2 diabetes (Kim et al., 2019; Huo et al., 2022). Considering MetS often precedes the development of cardiovascular and metabolic disease, it is not surprising that prior studies have extended these observations to individuals with MetS. For instance, several studies have reported significant relationships between time- and frequency-domain measures of HRV and the number and severity of MetS risk factors (Assoumou et al., 2010; Soares-Miranda et al., 2012; Carvalho et al., 2018), while others have reported significantly impaired HRV parameters among MetS patients compared to matched control subjects (Gehi et al., 2009). Likewise, others have examined MetS related changes in baroreflex control, exercise pressor responses, and metaboreflex sensitivity. Collectively, these studies would indicate that the blood pressure responses to handgrip exercise (Limberg et al., 2014; Endukuru et al., 2020) and metaboreflex activation (Limberg et al., 2014; Milia et al., 2015) are not significantly altered in individuals with MetS, while baroreflex sensitivity is attenuated (Grassi et al., 2005; Endukuru et al., 2020; Dutra-Marques et al., 2021). However, while each of these studies significantly contributes to the overall body of the literature, some elements of these reports warrant further consideration.

First, despite not reaching statistical significance, it is notable that these studies consistently reported net increases in the blood pressure responses to both handgrip exercise (Limberg et al., 2014; Endukuru et al., 2020) and metaboreflex activation (Limberg et al., 2014; Milia et al., 2015) in individuals with MetS. Considering this, and the fact that others have reported exaggerated blood pressure responses during locomotor exercise in individuals with MetS (Dutra-Marques et al., 2021), more research is needed to comprehensively evaluate MetS-related changes in exercise pressor and metaboreflex responses. Second, the influence of the hypertensive component of MetS on these responses is still relatively unclear. Dutra-Marques et al. (2021) reported evidence that individuals with a non-hypertensive MetS phenotype demonstrate exaggerated exercise pressor responses during cardiopulmonary exercise testing, suggesting that MetS-related changes in exercise pressor responses can occur independently from resting hypertension. However, this study did not evaluate metaboreflex responses, and the exclusion of hypertensive individuals limits the ability to evaluate the relative influence of resting blood pressure within a hypertensive MetS phenotype.

To that end, the aims of this study were twofold. First, we aimed to directly compare the hemodynamic responses to isometric handgrip and metaboreflex activation between individuals with MetS and control subjects matched for age, biological sex, race, and ethnicity. We expected to observe significantly exaggerated blood pressure and heart rate (HR) responses to handgrip exercise and metaboreflex activation in the MetS group compared to control participants. Secondly, we aimed to determine the potential mediating role of resting blood pressure in explaining these differences. Considering the known association between resting hypertension and exaggerated exercise pressor responses (Delaney et al., 2010; Greaney et al., 2014), and considering that treatment with angiotensin receptor blockers (ARBs) has been shown to attenuate exercise pressor responses in individuals with MetS (Nashar et al., 2004), we expected each of the significant differences in exercise pressor responses and metaboreflex responses to be abated after adjusting for resting blood pressure. If true, this would support the notion that 1) exercise pressor and metaboreflex responses are exaggerated in individuals with MetS and 2) the presence of comorbid hypertension significantly contributes to these differences. This information would add to the understanding of cardiovascular dysregulation in MetS and inform the development of pharmacological and non-pharmacological mitigation strategies in this population.

## Materials and methods

### Study participants

A total of 132 individuals were initially recruited for participation in this study, all of whom were prescreened for MetS. Of these 132 individuals, seventy individuals completed both study visits, eighteen of whom met the criteria for MetS, defined as having any combination of three of the following cardiometabolic risk factors (Grundy et al., 2005): 1) waist circumference (WC) ≥102 cm for males or ≥88 cm for females (≥80 cm for Asian females), 2) resting systolic blood pressure (SBP) ≥130 mmHg or diastolic blood pressure (DBP) ≥85 mmHg, 3) fasting blood glucose (FBG) ≥100 mg/dL or HbA1C ≥ 5.7%, 4) fasting HDL cholesterol (HDL-C) <50 mg/dL in females or <40 mg/dL in males, or 5) fasting triglycerides (TRG) ≥150 mg/dL. Furthermore, any individuals prescribed medications to control any of these risk factors (*n* = 4 in the final analysis) were also counted as expressing that risk factor. Of these eighteen subjects, thirteen were able to be matched to control subjects. Control subjects presented with ≤2 cardiometabolic risk factors, were not taking any medications known to affect cardiometabolic risk factors, and were matched to subjects in the MetS group by age (mean difference between matched pairs = 2 ± 5 years), biological sex assigned at birth, race, and ethnicity. This matching procedure resulted in four matched pairs of non-Hispanic Black/African American (B/AA) males, three matched pairs of non-Hispanic B/AA females, three matched pairs of non-Hispanic White males, one matched pairs of non-Hispanic White females, one matched pair of Hispanic White females, and one matched pair of non-Hispanic Asian females. All other subject demographics are presented in Table 1. All protocols used in this study were approved by the University of Southern Mississippi Institutional Review Board (IRB # 22-1012), and all participants provided written informed consent.

**TABLE 1:** Participant demographics.

	MetS	Control	*t*	*p*
Male/Female (*n*)	6/7	6/7	—	—
Age (years)	28 ± 12	30 ± 13	0.401	0.34
MVC (kg)	45.6 ± 14.0	37.3 ± 11.9	1.646	0.05
Height (cm)	172.9 ± 10.6	168.3 ± 10.8	1.107	0.14
Weight (kg)	106.7 ± 39.2	74.7 ± 21.4	2.588	<0.01*
BMI (kg/m^2^)	35.0 ± 10.1	26.1 ± 6.6	2.653	<0.01*
WC (cm)	109.4 ± 24.3	86.3 ± 14.8	2.933	<0.01*
FBG (mg/dL)	98 ± 13	88 ± 6	2.468	0.01*
HbA1C (%)	5.29 ± 0.66	4.97 ± 0.27	1.640	0.05
OGT (mg/dL)	107 ± 27	107 ± 14	0.054	0.47
RSBP (mmHg)	118 ± 12	115 ± 10	0.841	0.20
RDBP (mmHg)	83 ± 12	75 ± 9	1.974	0.03*
RHR (bpm)	70 ± 15	63 ± 10	1.391	0.08
HDL-C (mg/dL)	36 ± 11	50 ± 11	3.212	<0.01*
LDL-C (mg/dL)	103 ± 15	88 ± 22	1.595	0.06
TRG (mg/dL)	211 ± 219	113 ± 89	1.501	0.07
TC (mg/dL)	161 ± 48	160 ± 29	0.099	0.46
Total Body Fat (%)	34.6 ± 6.8	28.5 ± 6.7	2.299	0.01*
Fat Mass (kg)	39.6 ± 18.6	20.2 ± 9.6	3.340	<0.01*
Fat-Free Mass (kg)	70.2 ± 20.2	51.1 ± 13.2	2.856	<0.01*
MetS_index_	0.74 ± 1.06	−0.52 ± 0.68	3.620	<0.01*

MetS, metabolic syndrome group; *t*, absolute value of the *t*-statistic; *p*, *p*-value; BMI, body mass index; WC, waist circumference; FBG, fasting blood glucose; OGT, 2-h oral glucose tolerance; RSBP, resting systolic blood pressure; RDBP, resting diastolic blood pressure; RHR, resting heart rate; LDL-C, fasting LDL-cholesterol; HDL-C, fasting HDL-cholesterol; TRG, fasting triglyceride concentration; TC, total cholesterol; *, statistically significant difference between groups (*p*< 0.05). Data presented as Mean ± standard deviation, *(sample size)*

### Experimental design

Subjects in this study completed two research visits, the first of which served as a cardiometabolic prescreening. Subjects arrived at the prescreening visit at least 8 hours postprandial, which included abstention from caffeine and prescription or over-the-counter medications/supplements, and having avoided physical exertion (i.e., exercise) for 24 hours prior. Upon arrival, subjects underwent anthropometric and body composition assessments, followed by the assessment of FBG, fasting blood lipid concentrations, and oral glucose tolerance (OGT). The second visit served as an assessment of resting cBRS and reflex cardiovascular control. Similar to the first visit, subjects arrived at the second visit at least 8 hours postprandial, including caffeine, and having abstained from over-the-counter medications and alcohol for 12 hours and intense physical activity for 24 hours prior. The second visit included assessments of the hemodynamic responses to handgrip exercise and metaboreflex activation.

### Anthropometrics and body composition assessments

WC was evaluated using a standard spring-loaded aluminum tape measure. In brief, the “zero” marking of the tape measure was placed below the umbilicus and traversed around the torso horizontally with minimal tension (to not compress the skin/subcutaneous adipose layer) at the level of the iliac crest as suggested for the assessment of MetS (Grundy et al., 2005). Body composition was assessed using bioimpedance spectroscopy (BIS; SFB7, ImpediMed^®^, Carlsbad, CA, United States). BIS operates by introducing an array of electrical currents through the body and measuring the body’s opposition to these currents, which can be used to estimate total body fat and fat-free mass from estimates of total body water (Graybeal et al., 2023). This assessment required subjects to lay supine for ∼ 5 minutes while electrodes placed on the hands and feet (∼5 cm apart) delivered and recorded bioelectrical resistance and reactance which subsequently produced estimates of body composition. BIS has been shown to be a valid and reliable assessment of body composition in humans (Esco et al., 2019).

### Fasting blood glucose and lipids

FBG and lipids were collected using a point-of-care cholesterol analyzer (Cholestech LDX, Abott, Abbott Park, IL), which has demonstrated accuracy compared to other laboratory methods (Issa et al., 1996; Dale et al., 2008). After a minimum 8-h fast from food, beverage, supplements, and medications, ∼40 µL of capillary blood was collected via fingerstick using lithium heparin-lined capillary pipettes and applied to a pre-packaged cartridge. This cartridge provided measures of LDL cholesterol (LDL-C; %CV: 3.8–4.9), HDL-C (%CV: 3.3–4.9), total serum cholesterol (TC; %CV: 2.4–2.5), TRG (%CV: 1.6–3.6), and blood glucose (%CV: 4.5–6.2). LDL-C was calculated as:

$$LDL\;–C=TC\;–\;HDL\;–C\;–\;\left(TRG/5\right)$$

The cholesterol analyzer was calibrated before each new batch of cassettes (based on Lot #), as recommended by the manufacturer, using two (high/low) multianalyte control solutions. All values produced during calibration were within the expected ranges outlined by the manufacturer. Daily, and prior to testing, the analyzer’s optical scanner was calibrated using a standardized optical control cassette. Notably, this reader would not record TRG values above 650 mg/dL or below 45 mg/dL. Two individuals with MetS returned TRG readings of >650 mg/dL, which were recorded as 650 mg/dL, and four individuals in the control group produced TRG values of <45 mg/dL, which were recorded as 45 mg/dL. Therefore, TRG were likely under- and overestimated in these individuals, respectively. Moreover, this analyzer is unable to produce HDL-C measurements when TRG readings are >650 mg/dL (due to accuracy concerns) and thus, HDL-C was not recorded for the subjects with TRG >650 mg/dL. However, the absence of an HDL-C value did not change the MetS classification for these subjects. In addition, the cholesterol analyzer does not record HDL-C values below 15 mg/dL. One individual in the MetS group returned HDL-C values < 15 mg/dL, which was recorded as 15 mg/dL. Thus, HDL-C was likely overestimated in this subject. Because LDL-C is calculated from TC, TRG, and HDL-C, subjects that had TRG and HDL-C measurements outside of the analyzer’s detectable range (*n* = 9) did not receive measurements of LDL-C.

Glycated hemoglobin (HbA1C) was recorded using a second automated HbA1C reader (A1CNow+, pts diagnostics, Whitestown, IN) as a supplement to FBG. This HbA1C analyzer has been shown to be accurate when compared to laboratory-grade reference methods (Jiang et al., 2014). To measure HbA1C, ∼5 µL of capillary blood was collected from the finger using an extraction tube which was then inserted into a pre-packaged sample dilution tube. After adequate mixing of the dilution tube, the sample was placed into a single use cartridge which was subsequently inserted into the HbA1C analyzer. During each test, the HbA1C analyzer performs over 50 internal quality assurance checks that assess hardware, software, and reagent errors. In the instance that errors are detected, the analyzer does not produce an estimate of HbA1C. Thus, all measurements of HbA1C in this study passed all quality assurance tests.

### Oral glucose tolerance

In addition to FBG, glucose control was also evaluated using a standard oral glucose tolerance (OGT) test. Subjects were instructed to consume ≥150 g/day of carbohydrates for at least 3 days prior to the collection of data, which was verified using electronic food records. Each OGT test began with a baseline collection of capillary blood glucose (GK + Glucose & Ketone Meter, Keto-Mojo, Napa, CA, USA), in duplicate, followed by the ingestion of a 250 mL solution of water and 75 g glucose (dextrose; Dextrose Powder, NOW^®^, Bloomingdale, IL, Lot #3261408), which was confirmed via third-party testing (Informed Sport, Lexington, KY, United States). Blood glucose was then collected every 30 minutes, in duplicate, for 2 hours following ingestion of the carbohydrate bolus. Duplicate measurements were averaged at each timepoint to produce a final estimate of blood glucose and the final value recorded at the two-hour mark was recorded as the OGT value (mg/dL).

### Metabolic syndrome risk score (MetS_index_)

In addition to the more dichotomous classification of MetS or control, continuous MetS severity scores specific to sex and racial/ethnic groups were calculated for each individual using previously developed equations (Gurka et al., 2014). Each equation uses the most common MetS classification criteria which include WC, SBP, HDL-C, TRG, and FBG, where the inverse of HDL-C and the log of TRG are employed to ensure appropriate interpretation. Each individual score is interpreted as a Z-score where more positive scores represent an increased risk, and more negative scores represent a decreased risk. These equations have demonstrated “excellent” predictive ability for MetS classification and are highly associated with other proxies of disease risk (Gurka et al., 2014). Moreover, these equations were designed to account for age and existing conditions that may be controlled using medication, which was prominent in our study, that may have otherwise underestimated the value of the MetS component in question. DBP and HbA1C are not used in the equation due to issues of multicollinearity with other variables (i.e., SPB and FBG) although these were used in our study for MetS classification; where three subjects met the criterion based on these assessments (DBP: 7; HbA1C: 1). Lastly, there are no existing equations specific to Asian adults. Therefore, the non-Hispanic White female equation (Gurka et al., 2014) was used to calculate MetS severity scores for the two Asian females (MetS: 1; Con: 1) in our study.

### Cardiovascular reflex responses

In this study, cardiovascular reflex responses were defined as the hemodynamic responses to voluntary exercise and metaboreflex activation, which were evaluated using a standard handgrip and post-exercise circulatory occlusion (PECO) protocol. This protocol began with 2 minutes of isometric handgrip of the non-dominant arm, assigned at 35% of the subject’s maximal voluntary contraction (MVC), which was determined in triplicate prior to baseline data collection. Just prior to the end of the two-minute contraction period, a pneumatic pressure cuff (E20 Rapid Cuff Inflator, DE Hokanson, Bellevue, WA) was inflated around the upper arm to a suprasystolic pressure and maintained for 3 minutes. This three-minute period of PECO isolates (and exaggerates) the metaboreflex by trapping exercise related metabolic byproducts within the previously active forearm, promoting a sustained hypertensive response (Ichinose et al., 2004; Cui et al., 2008).

Throughout each assessment of cardiovascular function, cardiac rhythm was continuously recorded using a one-lead (lead I) electrocardiogram (ECG; PowerLab AD Instruments, Colorado Springs, CO), and beat-by-beat blood pressure was continuously recorded via finger photoplethysmography (Finapres NANO, AD Instruments, Colorado Springs, CO). The beat-by-beat blood pressure recorded from the finger was calibrated to brachial blood pressure values collected during the baseline period of each cardiovascular assessment. HR was calculated from each individual R-R interval, and mean arterial pressure (MAP) was calculated as the average of all samples (sampled at 1,000 Hz) within a single cardiac cycle. Similarly, SBP and DBP were calculated as the maximum and minimum points recorded within each individual cardiac cycle, respectively. These data were then used to identify the peak responses and relative changes (Δ) in SBP, DBP, MAP, and HR, as well as areas under the curve (for MAP only) observed during handgrip and PECO. The area under the curve for MAP, termed the blood pressure index (BPI; mmHg*sec), was also normalized to the time-tension index (kg*sec) for the HG period, providing a normalized BPI value (BPI_norm_; mmHg/kg).

### Statistical approach

Data were first visually inspected for normality and the presence of outliers using histograms and boxplots. Next, independent samples *t*-tests were used to test the following hypotheses: 1) individuals in the MetS group would demonstrate significantly augmented peak and relative hemodynamic responses to handgrip and PECO compared to the control group and 3) individuals in the MetS group would demonstrate significantly augmented BPI responses to handgrip and PECO compared to the control group. Analyses of covariance (ANCOVA) were then used to determine the influences of resting SBP (in the cases of SBP and HR responses) or resting DBP (in the cases of DBP, MAP, and BPI responses) in mediating any significant differences between groups. To determine if any potential changes in group differences were unique resting blood pressure, ANCOVA were also used to independently adjust for FBG and WC, and to adjust for all three covariates simultaneously. If resting blood pressure is, in fact, the primary contributor to exaggerated hemodynamic responses, hemodynamic responses should remain significantly different between groups after covarying for FBG and WC separately, but not after adjusting for resting blood pressure. Linear regression analyses were also used to examine the relationships between each dependent variable (each absolute and relative pressor response value) and the MetS_index_ score. All statistical analyses were performed using SPSS statistical analysis software (SPSS Statistics version 28, IBM Corp., Armonk, NY), and all data are presented as the mean ± standard deviation (SD). Of the thirteen individuals with MetS included in the final analysis, all thirteen completed the handgrip trials and twelve completed the PECO trial.

## Results

### Body composition and glucose control

As expected, subjects in the MetS group were significantly heavier (106.7 ± 39.2 kg vs. 74.7 ± 21.4 kg in MetS vs. control, respectively, *p* < 0.01), had a higher percentage of body fat (34.6% ± 6.8% vs. 28.5% ± 6.7% in MetS vs. control, respectively, *p* = 0.01), a higher waist circumference (109.4 ± 24.3 cm vs. 86.3 ± 14.8 cm in MetS vs. control, respectively, *p* < 0.01), carried more total fat mass (39.6 ± 18.6 kg vs. 20.2 ± 9.6 kg in MetS vs. control, respectively, *p* < 0.01) and fat-free mass (70.2 ± 20.2 kg vs. 51.1 ± 13.2 kg in MetS vs. control, respectively, *p* < 0.01), and tended to have a higher HbA1C (5.29% ± 0.66% vs. 4.97% ± 0.27% in MetS vs. control, respectively, *p* = 0.057) compared to controls (Table 1). Surprisingly, OGT was not different between groups (107 ± 27 mg/dL vs. 107 ± 14 mg/dL in MetS vs. control, respectively, *p* = 0.47). Nevertheless, DBP, BMI, and the MetS_index_ were all significantly higher in the MetS group compared to controls (all *p* ≤ 0.03), and HDL-C was significantly lower (*p* < 0.01), clearly demonstrating an increased cardiometabolic disease risk in the MetS group. A breakdown of MetS risk factors for each group (MetS vs. control) is also provided in Table 2.

**TABLE 2:** Metabolic syndrome (MetS) risk factor counts between groups.

	MetS (*n*)	Control (*n*)
WC	11	2
FBG/HbA1C	7	0
SBP/DBP	9	1
TRG	6	3
HDL	13	5
0 Risk Factors	0	7
1 Risk Factor	0	2
2 Risk Factors	0	4
3 Risk Factors	8	0
4 Risk Factors	2	0
5 Risk Factors	3	0

WC, waist circumference; FBG, fasting blood glucose; HbA1C, glycated hemoglobin; SBP, systolic blood pressure; DBP, diastolic blood pressure; TRG, triglycerides; HDL, high-density lipoprotein cholesterol; MetS, metabolic syndrome group.

### Unadjusted exercise pressor and metaboreflex responses

**FIGURE 1:**
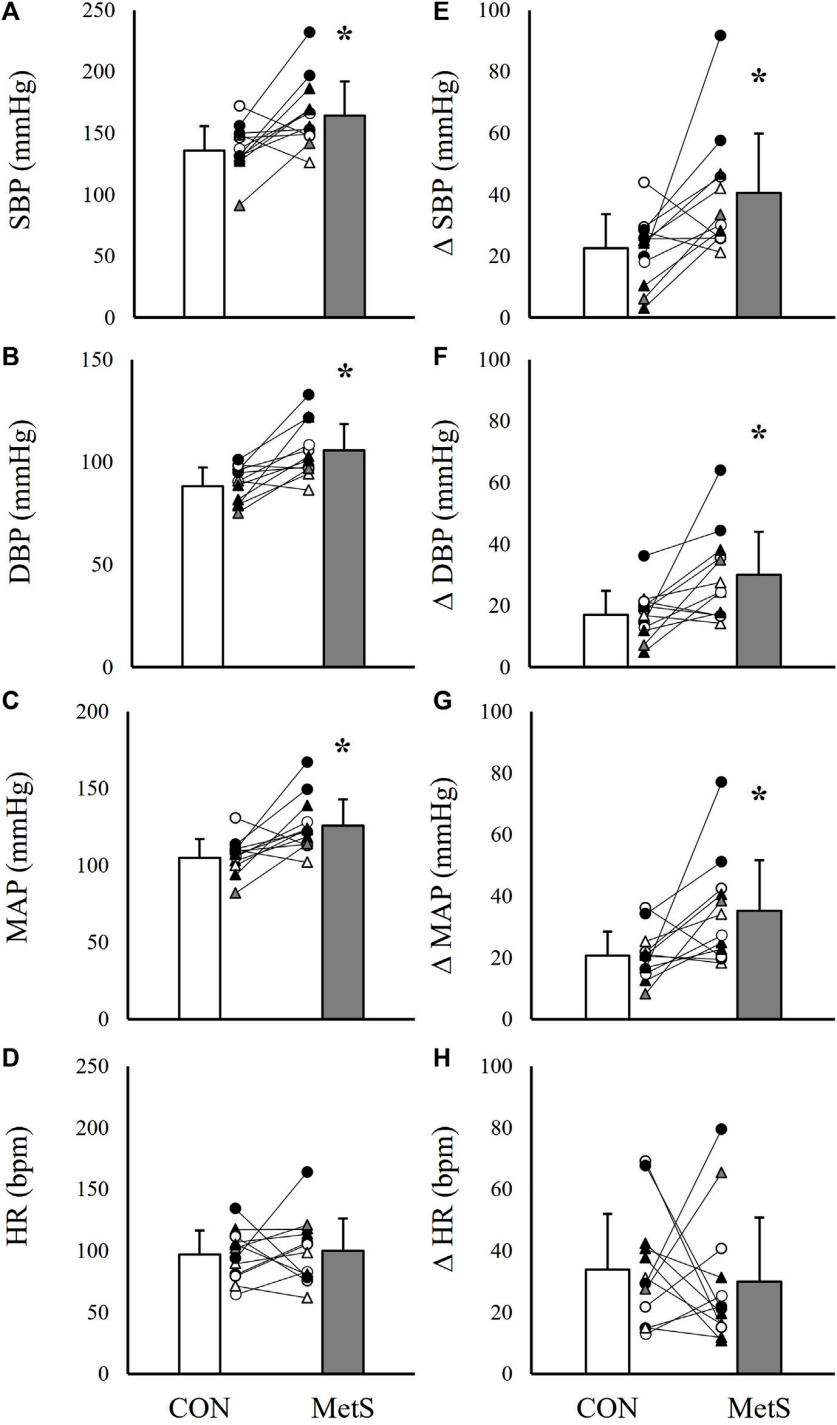
Absolute systolic blood pressure [SBP; **(A)**], diastolic blood pressure [DBP; **(B)**], mean arterial pressure [MAP; **(C)**], and heart rate [HR; **(D)**] responses to 2 minutes of handgrip exercise (35% MVC) compared between individuals with (MetS) and without (CON) metabolic syndrome. Panels **(E–H)** depict group differences in the relative change scores (Δ) for each value. Black filled symbols represent Black/African American participants, white filled symbols represent White participants, gray filled symbols represent Asian participants, circles represent male participants, and triangles represent female participants. Lines connecting raw data points indicate participants matched for age, biological sex, race, and ethnicity. Data presented as mean ± standard deviation, * indicates a statistically significant difference compared to the control group (*p* < 0.05).

**FIGURE 2:**
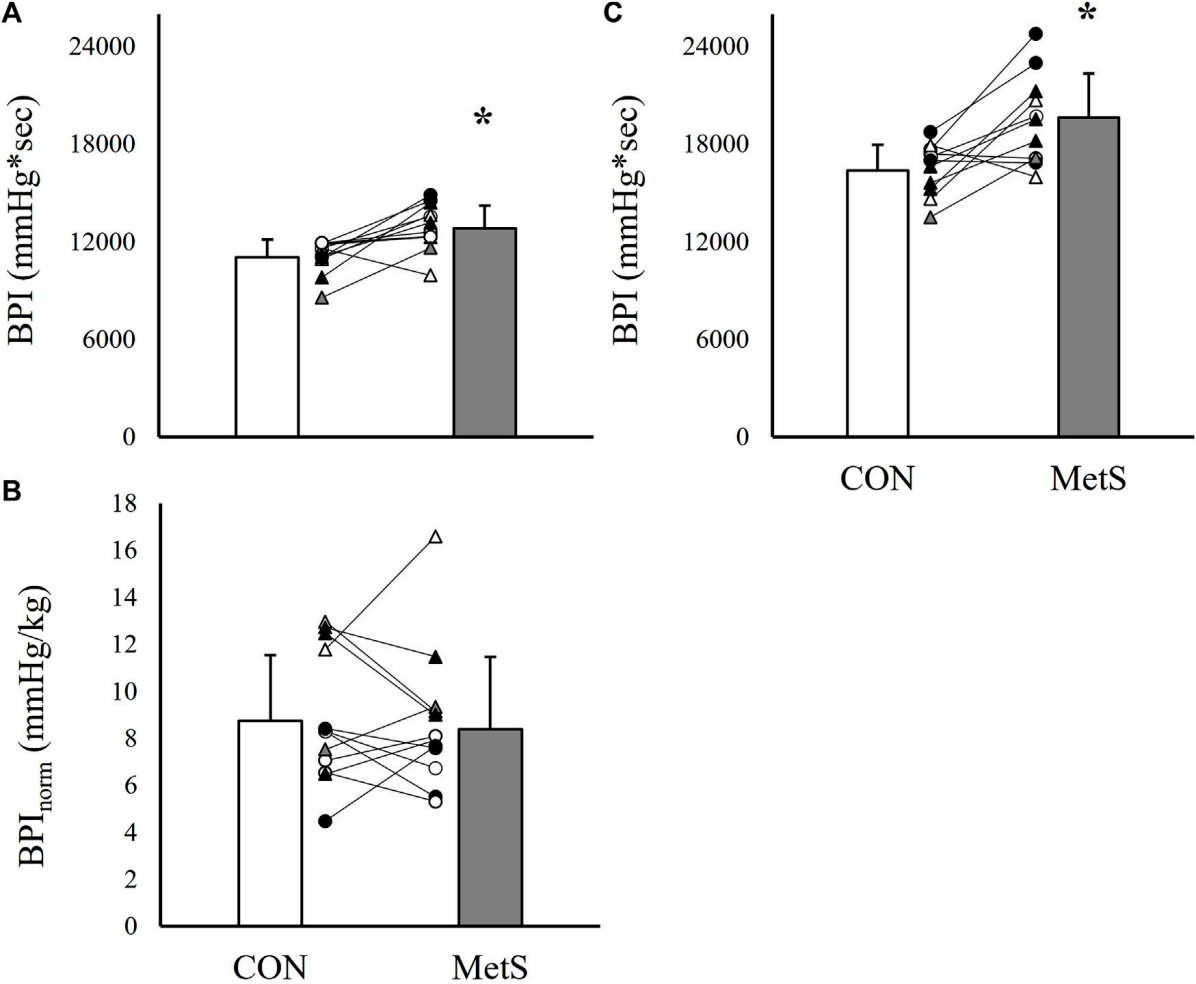
Total area under the curve for mean arterial pressure [BPI; **(A)**] and BPI normalized to the time-tension index [BPI_norm_; **(B)**] recorded during 2 minutes of handgrip exercise (35% MVC) compared between individuals with (MetS) and without (CON) metabolic syndrome. Panels **(C)** represent BPI responses recorded during 3 minutes of post-exercise circulatory occlusion. Black filled symbols represent Black/African American participants, white filled symbols represent White participants, gray filled symbols represent Asian participants, circles represent male participants, and triangles represent female participants. Lines connecting raw data points indicate participants matched for age, biological sex, race, and ethnicity. Data presented as mean ± standard deviation, * indicates a statistically significant difference compared to the control group (*p* < 0.05).

**FIGURE 3:**
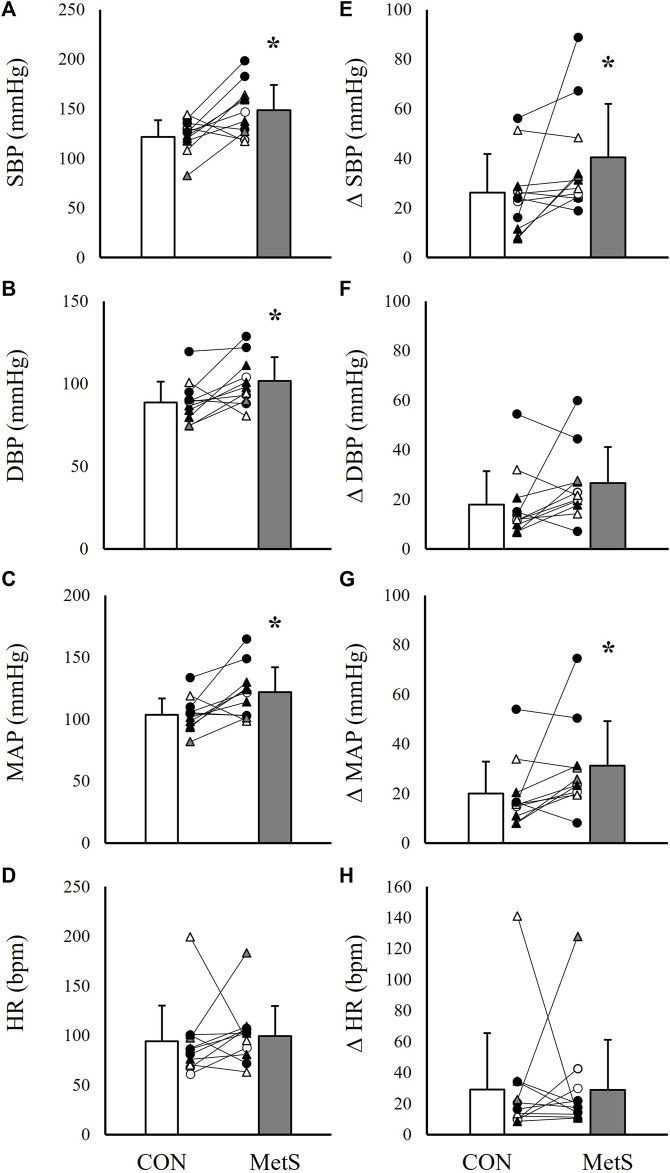
Absolute systolic blood pressure [SBP; **(A)**], diastolic blood pressure (DBP; **(B)**, mean arterial pressure [MAP; **(C)**], and heart rate [HR; **(D)**] responses to 3 minutes of post-exercise circulatory occlusion compared between individuals with (MetS) and without (CON) metabolic syndrome. Panels **(E–H)** depict group differences in the relative change scores (Δ) for each value. Black filled symbols represent Black/African American participants, white filled symbols represent White participants, gray filled symbols represent Asian participants, circles represent male participants, and triangles represent female participants. Lines connecting raw data points indicate participants matched for age, biological sex, race, and ethnicity. Data presented as mean ± standard deviation, * indicates a statistically significant difference compared to the control group (*p* < 0.05).

Results indicated significantly exaggerated peak and relative pressor responses to both HG and PECO in the MetS group compared to controls (Figures 1–3). Specifically, peak SBP responses were elevated by 20.6% (*p* < 0.01; Figure 1A), peak DBP responses by 19.3% (*p* < 0.01; Figure 1B), peak MAP responses by 20.0% (*p* < 0.01; Figure 1C), and BPI responses by 16.2% (*p* < 0.01; Figure 2A) in the MetS group compared to controls during handgrip exercise. This corresponded to a 78.3% higher ΔSBP response (*p* < 0.01; Figure 1E), a 76.5% higher ΔDBP response (*p* < 0.01; Figure 1F), and a 66.7% higher ΔMAP response (*p* < 0.01; Figure 1G) in the MetS group compared to controls during handgrip. Peak HR responses were 3.1% higher in the MetS group compared to controls, however, this comparison failed to reach statistical significance [mean diff = 2.9 (CI: 15.9/21.7), *p* = 0.37; Figures 1D–H]. No significant difference was observed for BPI_norm_ during handgrip exercise [8.39 ± 3.09 mmHg/kg vs. 8.73 ± 2.81 mmHg/kg in MetS vs. control, respectively, mean diff = −0.35 (CI: −2.74/2.04), *p* = 0.38; Figure 2B].

When the peak pressor responses to PECO were evaluated, results indicated similarly elevated peak pressor responses in the MetS group compared to controls. As in the handgrip trials, SBP was elevated by 18.8% (*p* = 0.01; Figure 3A), DBP by 14.6% (*p* = 0.01; Figure 3B), MAP by 17.3% (*p* < 0.01; Figure 3C), and BPI by 19.9% (*p* < 0.01; Figure 2C) in the Mets group compared to controls. This also corresponded to significantly exaggerated ΔSBP (53.8% higher in MetS, *p* = 0.03) and ΔMAP (55.0% higher in MetS, *p* = 0.04; Figures 3E–G) responses in the MetS group compared to controls during PECO. No differences were observed for HR responses between groups (Figures 3D–H, all *p* ≥ 0.30).

### Adjusted exercise pressor and metaboreflex responses

Results from the covariate analyses are presented in Table 3. When adjusting for resting blood pressure, peak SBP (*F* = 8.415, *p* < 0.01), DBP (*F* = 11.899, *p* < 0.01), and MAP responses (*F* = 10.312, *p* < 0.01), as well as ΔSBP (*F* = 8.218, *p* < 0.01), ΔDBP (*F* = 10.317, *p* < 0.01), ΔMAP (*F* = 9.158, *p* < 0.01), and BPI responses (*F* = 10.612, *p* < 0.01) remained significantly exaggerated in the MetS group. Interestingly, however, these hemodynamic responses to handgrip also remained significantly exaggerated in MetS after independently adjusting for FBG (all *p* < 0.01), and all but ΔMAP (*F* = 4.247, *p* = 0.051) remained significantly exaggerated in Mets after independently adjusting for WC (all *p* ≤ 0.04). Likewise, peak SBP (*F* = 6.003, *p* = 0.02), DBP (*F* = 5.190, *p* = 0.03), and MAP (*F* = 6.501, *p* = 0.01), as well as ΔMAP (*F* = 4.475, *p* = 0.04), and BPI (*F* = 11.658, *p* < 0.01) responses to PECO remained significantly exaggerated in the MetS group after adjusting for resting blood pressure, and these same responses, with the exception of ΔMAP (*F* = 2.659, *p* = 0.11), remained significantly exaggerated after independently adjusting for FBG (all *p* ≤ 0.03). Only peak SBP (*F* = 5.592, *p* = 0.02) and BPI responses (*F* = 6.355, *p* = 0.02) to PECO remained significantly exaggerated in MetS after independently adjusting for WC.

**TABLE 3:** Influence of metabolic syndrome on exercise pressor and metaboreflex responses after adjusting for resting blood pressure, fasting blood glucose, and waist circumference.

Controlling for	RBP			FBG			WC			RBP, FBG, and WC		
	*Mean Diff*	*CI (95%)*	*Sig*	*Mean Diff*	*CI (95%)*	*Sig*	*Mean Diff*	*CI (95%)*	*Sig*	*Mean Diff*	*CI (95%)*	*Sig*
*Handgrip*												
SBP_peak_ (*mmHg*)	28 ± 10	8/48	<0.01*	29 ± 10	9/49	<0.01*	29 ± 12	4/53	0.02*	29 ± 13	3/55	0.03*
ΔSBP (*mmHg*)	18 ± 6	5/31	<0.01*	19 ± 6	6/32	<0.01*	16 ± 8	0/31	0.04*	15 ± 8	−2/31	0.08
DBP_peak_ (*mmHg*)	17 ± 5	7/27	<0.01*	17 ± 5	8/26	<0.01*	14 ± 5	3/24	0.01*	14 ± 6	3/26	0.01*
ΔDBP (*mmHg*)	16 ± 5	6/26	<0.01*	13 ± 5	4/23	<0.01*	13 ± 5	1/24	0.02*	14 ± 6	3/26	0.02*
MAP_peak_ (*mmHg*)	21 ± 7	8/35	<0.01*	21 ± 6	8/33	<0.01*	18 ± 7	3/32	0.01*	18 ± 8	3/34	0.02*
ΔMAP (*mmHg*)	17 ± 6	5/28	<0.01*	15 ± 5	4/26	<0.01*	13 ± 6	0/25	0.05	14 ± 6	1/27	0.03*
HR_peak_ (*mmHg*)	2 ± 9	−17/20	0.85	2 ± 9	1/17	0.87	−5 ± 11	−28/17	0.62	−2 ± 11	−26/21	0.84
ΔHR (*bpm*)	−5 ± 7	−20/10	0.49	−5 ± 8	−21/11	0.52	−11 ± 9	−30/8	0.23	−8 ± 9	−27/12	0.43
BPI (mmHg*sec)	1793	655/2,932	<0.01*	1792	749/2,834	<0.01*	1,676 ± 598	439/2,912	0.01*	1,688	353/3,023	0.01*
	±550			±504						±642		
*PECO*												
SBP_peak_ (*mmHg*)	26 ± 11	4/49	0.02*	25 ± 11	3/47	0.03*	31 ± 13	4/58	0.02*	30 ± 14	1/60	0.04*
ΔSBP (*mmHg*)	15 ± 8	−1/31	0.07	14 ± 6	−3/30	0.09	16 ± 10	−4/35	0.11	13 ± 10	−8/35	0.20
DBP_peak_ (*mmHg*)	14 ± 6	1/27	0.03*	12 ± 5	1/23	0.03*	10 ± 7	−4/24	0.14	12 ± 7	−2/26	0.08
ΔDBP (*mmHg*)	12 ± 6	0/25	0.06	8 ± 6	−4/20	0.18	9 + 7	−6/24	0.21	12 ± 7	−3/26	0.10
MAP_peak_ (*mmHg*)	20 ± 8	4/36	0.01*	17 ± 7	3/31	0.02*	17 ± 9	−1/35	0.06	19 ± 9	1/38	0.03*
ΔMAP (*mmHg*)	15 ± 7	0/29	0.04*	11 + 6	−3/24	0.11	11 ± 8	−6/28	0.18	14 ± 8	−3/31	0.10
HR_peak_ (*bpm*)	5 ± 14	−24/34	0.70	6 ± 14	−23/35	0.66	14 ± 17	−20/48	0.40	18 ± 18	−20/56	0.32
ΔHR (*mmHg*)	0 ± 14	−31/30	0.98	0 ± 15	−30/30	0.98	10 ± 17	−25/46	0.55	14 ± 19	−25/53	0.45
BPI (mmHg*sec)	3,437	1,343/5,530	<0.01*	3,125	1,257/4,993	<0.01*	2,801	490/5,112	0.02*	3,078	674/5,482	0.01*
	±1,007			±898			±1,111			±1,149		

RBP, resting blood pressure; FBG, fasting blood glucose; WC, waist circumference; SBP, systolic blood pressure; DBP, diastolic blood pressure; MAP, mean arterial pressure; HR, heart rate; BPI, blood pressure index; Δ, delta score; PECO, post-exercise circulatory occlusion *Mean Diff*, mean difference ±standard error around the mean; *CI*, 95% confidence intervals; *Sig*, *p*-value.

When all three covariates were included in the model, peak SBP (*F* = 5.256, *p* = 0.03), DBP (*F* = 6.471, *p* = 0.01), and MAP (*F* = 5.867, *p* = 0.02), as well as ΔDBP (*F* = 6.396, *p* = 0.02), ΔMAP (*F* = 4.896, *p* = 0.03), and BPI responses (*F* = 6.913, *p* = 0.01) to handgrip exercise remained significantly exaggerated in the MetS group compared to controls. Likewise, peak SBP (*F* = 4.711, *p* = 0.04), peak MAP (*F* = 4.902, *p* = 0.03), and BPI (*F* = 7.184, *p* = 0.01) responses to PECO also remained significantly exaggerated in MetS vs. controls after adjusting for all three covariates.

**FIGURE 4:**
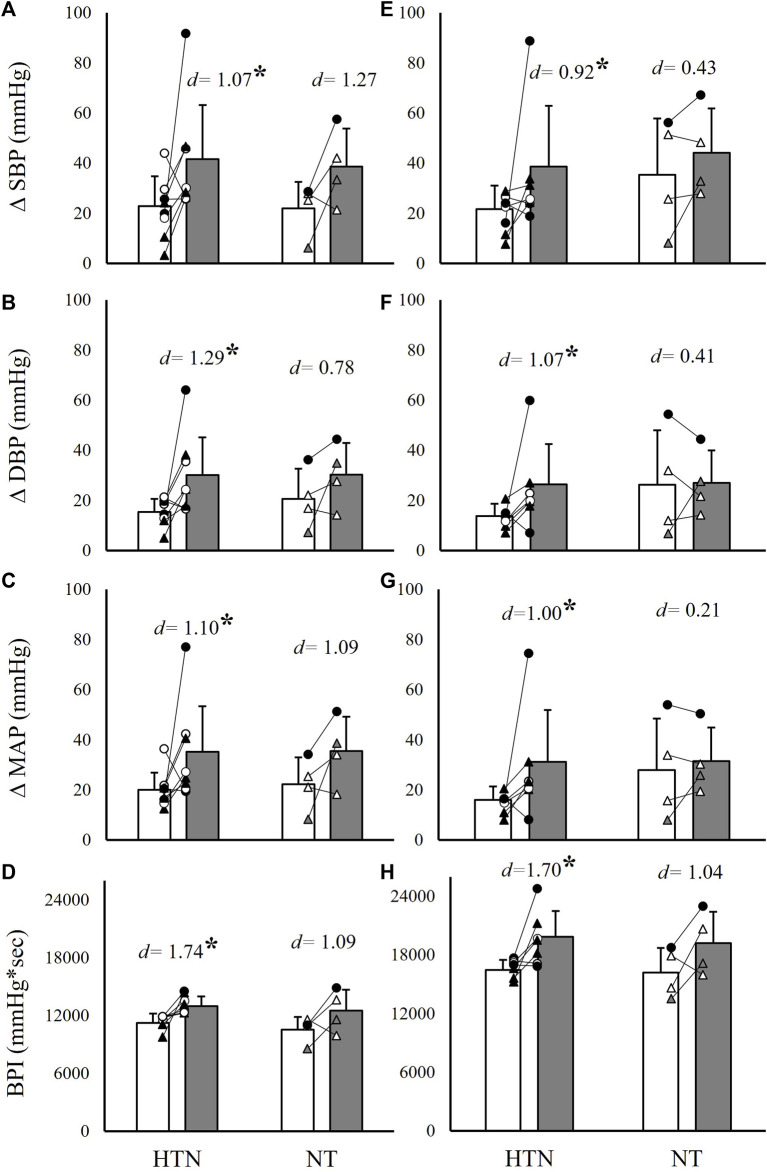
Absolute systolic blood pressure (SBP), diastolic blood pressure (DBP), mean arterial pressure (MAP), and cumulative blood pressure (BPI) responses to 2 minutes of isometric handgrip exercise [35% MVC; panels **(A–D)**] and 3 minutes of post-exercise circulatory occlusion [panels **(E–H)**] compared between individuals with (MetS) and without (CON) metabolic syndrome. Within each panel, group comparisons are further divided into hypertensive (HPTN) and normotensive (NT) MetS subgroups. Black filled symbols represent Black/African American participants, white filled symbols represent White participants, gray filled symbols represent Asian participants, circles represent male participants, and triangles represent female participants. Lines connecting raw data points indicate participants matched for age, biological sex, race, and ethnicity. Data presented as mean ± standard deviation; * indicates a statistically significant difference compared to the control group (*p* < 0.05); *d* indicates Cohen’s *d*.

**FIGURE 5:**
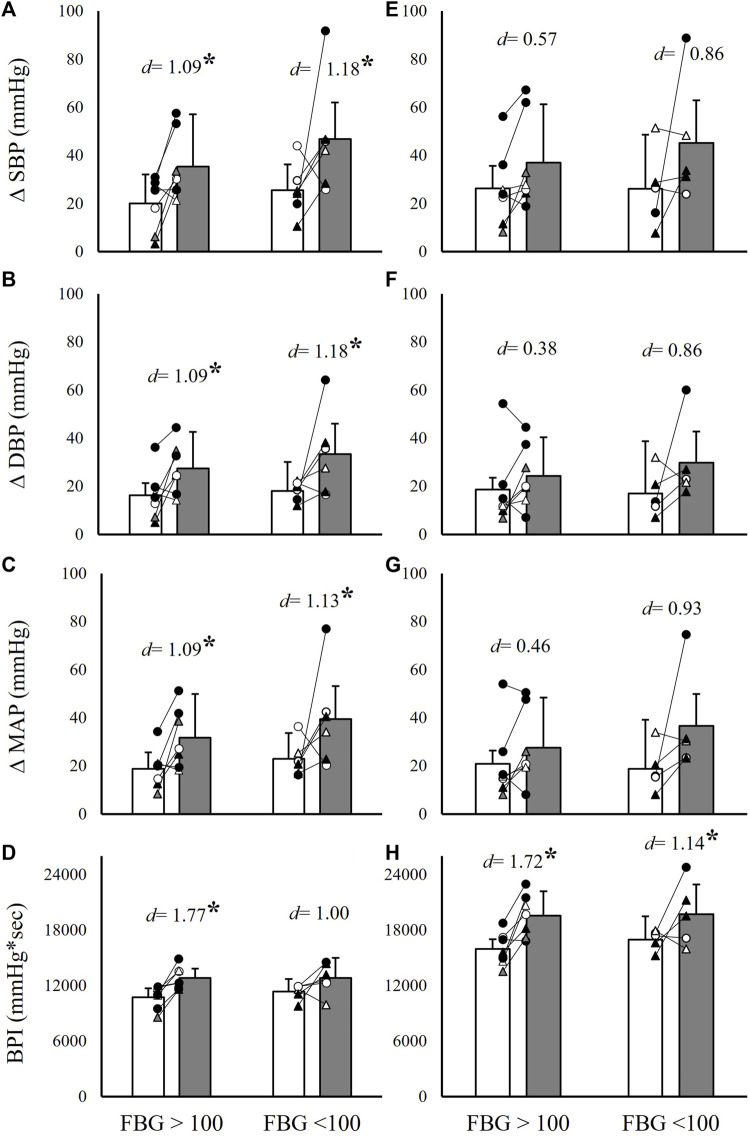
Absolute systolic blood pressure (SBP), diastolic blood pressure (DBP), mean arterial pressure (MAP), and cumulative blood pressure (BPI) responses to 2 minutes of isometric handgrip exercise [35% MVC; panels **(A–D)**] and 3 minutes of post-exercise circulatory occlusion [panels **(E–H)**] compared between individuals with (MetS) and without (CON) metabolic syndrome. Within each panel, group comparisons are further divided into hyperglycemic (FBG>100) and non-hyperglycemic (FBG<100) MetS subgroups. Black filled symbols represent Black/African American participants, white filled symbols represent White participants, gray filled symbols represent Asian participants, circles represent male participants, and triangles represent female participants. Lines connecting raw data points indicate participants matched for age, biological sex, race, and ethnicity. Data presented as mean ± standard deviation; * indicates a statistically significant difference compared to the control group (*p* < 0.05); *d* indicates Cohen’s *d*.

To further evaluate differences between hypertensive and non-hypertensive MetS phenotypes, as well as hyperglycemic and non-hyperglycemic phenotypes, the MetS group was separated by the presence or absence of hypertension and impaired fasting glucose, respectively. This resulted in nine hypertensive individuals with MetS vs. four normotensive individuals with MetS, and seven individuals with MetS and impaired fasting glucose vs. six individuals with MetS without impaired fasting glucose. Independent samples *t*-tests were then used to compare ΔSBP, ΔDBP, ΔMAP, and BPI between the MetS group and matched controls within these subgroups. Results indicated significantly exaggerated ΔSBP (82.6% and 77.3% higher in MetS, *p* ≤ 0.04), ΔDBP (100.0% and 85.7% higher in MetS, *p* ≤ 0.02), ΔMAP (75.0% and 93.8% higher in MetS, *p* ≤ 0.03), and BPI (15.2% and 20.6% higher in MetS, *p* < 0.01) responses to handgrip and PECO, respectively, in the hypertensive MetS group compared to matched controls (*n* = 9; Figure 4). Likely due to the small sample size (*n* = 4), comparisons between the non-hypertensive MetS group and matched controls failed to reach statistical significance, but demonstrated comparable effect sizes (Cohen’s *d*) for differences in handgrip responses relative to the comparisons in the hypertensive MetS group (Figure 4). In contrast, both the hyperglycemic (*n* = 7) and non-hyperglycemic (*n* = 6) MetS groups demonstrated significantly exaggerated ΔSBP (56.0% and 126.3% higher in MetS, respectively, *p* ≤ 0.03), ΔDBP (57.9% and 106.7% higher in MetS, respectively, *p* ≤ 0.03), and ΔMAP (54.5% and 94.7% higher in MetS, respectively, *p* ≤ 0.04) responses to handgrip exercise, and significantly exaggerated BPI responses to PECO (23.5% and 16.4% higher in MetS, respectively, *p* ≤ 0.03), compared to matched controls (Figure 5). Of note, the MetS group was not separated by WC, as all but two individuals in the MetS group had elevated WC values.

### Linear regression analyses

Results from linear regression analyses are presented in Table 4. MetS_index_ was significantly associated with BPI during PECO (*R* ^2^ = 0.203, *β* = 1,132.23, *p* = 0.02), but no other significant relationships were observed between the MetS_index_ and hemodynamic responses.

**TABLE 4:** Linear regression results for handgrip and PECO trials and MetS_index_.

	ΔSBP (mmHg)	ΔDBP (mmHg)	ΔMAP (mmHg)	BPI (mmHg*Sec)	BPI_norm_ (mmHg/kg)	ΔHR (bpm)
*Handgrip*						
*R* ^2^	0.067	0.095	0.085	0.143	0.101	0.029
β	4.263	3.646	3.929	529.025	−0.848	2.994
*Sig*	*0.20*	*0.12*	*0.14*	*0.05*	*0.11*	*0.40*
*PECO*						
*R* ^2^	0.030	0.054	0.058	0.203	—	0.079
β	3.148	3.060	3.625	1,132.227	—	−8.742
*Sig*	*0.41*	*0.27*	*0.25*	0.02*	—	*0.18*

SBP, systolic blood pressure; DBP, diastolic blood pressure; MAP, mean arterial pressure; BPI, blood pressure index; HR, heart rate; *Sig*, *p*-value; *R* ^2^, results from a linear regression between metabolic syndrome score and the listed dependent variable; β, unstandardized beta coefficient; PECO, post-exercise circulatory occlusion.

## Discussion

This study tested the general hypotheses that 1) cardiovascular reflex responses would be exaggerated in individuals with MetS compared to matched control subjects and 2) these differences would be largely explained by differences in resting blood pressure. Our findings only partially support these hypotheses. Specifically, individuals with MetS demonstrated significantly augmented blood pressure responses to both handgrip exercise and metaboreflex activation, which supports the primary hypothesis. However, these responses remained significantly exaggerated in individuals with MetS even after adjusting for resting blood pressure, FBG, WC, and all three covariates combined. Ultimately, these findings provide strong evidence that 1) exercise pressor responses and metaboreflex responses are significantly exaggerated in MetS, and 2) these changes occur independent of differences in resting blood pressure, FBG, or WC. These findings provide novel insight into MetS related changes in cardiovascular function, as will be discussed in the following sections.

### Exercise pressor responses and metaboreflex activation in MetS

As noted previously, the magnitude of exercise pressor responses are known to be significantly exaggerated in some (Delaney et al., 2010; Muller et al., 2012; Greaney et al., 2014; Luck et al., 2017; Kim et al., 2019; Huo et al., 2022), but not all (Sterns et al., 1991; Negrão et al., 2001; Rondon et al., 2006; Dipla et al., 2010; Xing et al., 2015) cardiovascular or metabolic conditions. Because of this complexity, predicting how blood pressure would respond to voluntary exercise and metaboreflex activation in individuals with MetS can be challenging. Limberg and others approached this question in 2014 by comparing absolute and relative changes in MSNA and blood pressure during handgrip and PECO between MetS and control participants (Limberg et al., 2014). In this study, the relative changes in MSNA and blood pressure were not significantly different between groups, which would seem to suggest that neurocardiovascular reflex sensitivity is not altered in MetS. Our data would disagree with these findings. In the present study, both the absolute blood pressure responses and the relative changes in blood pressure during handgrip exercise and metaboreflex activation (Figures 1–3) were significantly exaggerated in the MetS group, and these differences persisted even after adjusting for resting blood pressure, FBG, and WC. One possible explanation for these contrasting findings could be differences in MetS phenotypes between the two studies. In the study by Limberg and others, participants in the MetS group demonstrated substantially lower FBG and TRG readings compared to individuals in MetS group in the present study. Therefore, it may be possible that different groupings of MetS risk factors elicit contrasting changes in cardiovascular function. However, the fact that covarying for FBG had no effect on our findings of exaggerated pressor responses in MetS would confound this notion. With that in mind, it may also be possible that the factors contributing to the development of MetS (i.e., behavioral or genetic factors) contribute more to the development of cardiovascular dysfunction than any single MetS risk factor. It is also worth noting that in the study by Limberg and others, absolute MAP responses to handgrip exercise were exaggerated in individuals with MetS compared to healthy controls, while the relative changes in blood pressure during handgrip and PECO only indicated non-significant net differences. Thus, while our conclusions regarding exercise pressor responses in MetS may differ, the trends in our data do not entirely disagree.

Specific factors that may have contributed to the observed increase in exercise pressor and metaboreflex responses in the MetS group of the present study may include impaired baroreflex control, augmented afferent receptor function or expression, or increases in central command. For instance, it is known that the baroreflex buffers systemic vascular responses during exercise pressor reflex activation (Kim et al., 2005a; Kim et al., 2005b; Ichinose et al., 2008; Ichinose et al., 2017; Mannozzi et al., 2022), and prior studies have demonstrated blunted baroreflex sensitivity in individuals with MetS (Grassi et al., 2005; Dutra-Marques et al., 2021). Therefore, altered baroreflex control may have contributed to the exaggerated pressor responses observed here. Likewise, others have identified contributions of TRPV1 channels (Smith et al., 2005; Wang et al., 2010; Yu and Wang, 2011), ASIC III channels (Xing et al., 2015), purinergic receptors (Wang et al., 2010; Greaney et al., 2014), and COX-2 expression (Smith et al., 2020) to exercise pressor responses in various cardiometabolic disease states. Considering that neither hypertension, FBG, nor central adiposity were able to explain the exaggerated exercise pressor and metaboreflex responses in individuals with MetS in the present study, it may be possible that changes in receptor expression occur secondary to the development of MetS. This notion is supported by the observations that 1) the exercise pressor reflex is significantly exaggerated in type 2 diabetic rats (Kim et al., 2019; Huo et al., 2022), 2) this adaptation occurs in parallel with disease progression (Grotle et al., 2019), and 3) acute glucose administration has no influence on exercise pressor reflex responses in non-diabetic rats (Huo et al., 2020). Thus, elevated glucose alone is not able to explain diabetes related cardiovascular adaptations, and instead, these adaptations are more likely explained by secondary adaptations to MetS development.

One finding from the present study that would support the argument that the magnitude of cardiovascular reflex responses to exercise (including both central command and the exercise pressor reflex) are not exaggerated in MetS is the lack of significant differences in BPI_norm_ responses during handgrip exercise (Figure 2B). Upon further analysis, the time-tension index (kg*sec) during handgrip was non-significantly elevated in the MetS group compared to controls (1,694.0 ± 561 kg*sec vs. 1,394.1 ± 481.2 kg*sec, respectively, *p* = 0.07), likely explained by differences in fat-free mass (Table 1). Although these differences in time-tension index were not statistically significant, the fact that cumulative blood pressure responses (BPI) were no longer different between the MetS and control groups after normalizing to time-tension index (on a subject-by-subject basis) could mean that absolute work output is the primary contributor to this augmented pressor response, as opposed to changes in neurocardiovascular sensitivity, *per se*. In other words, normalizing by relative intensity (35% MVC) revealed significantly exaggerated blood pressure responses (Figures 1, 2A), but normalizing by absolute work output (kg*sec) revealed nearly identical blood pressure responses (Figure 2B). This raises the rather philosophical question of which outcome is more relevant to real-world cardiovascular risk. On one hand, the observation that pressor responses are exaggerated during the same *relative* level of effort would suggest that individuals with MetS are predisposed to acute periods of exaggerated sympathetic activity during physical activity. On the other hand, however, the observation that pressor responses to the same level of *absolute* work are not different between groups would suggest that individuals with MetS are at no greater risk of acute periods of exaggerated sympathetic activity when performing the same general tasks as individuals without MetS (i.e., lifting a 20lb bag of groceries). Of course, this is a complex (and perhaps, somewhat controversial) perspective, as this would challenge the traditional use of relative handgrip intensities as a method of normalizing sympathetic stimuli. Furthermore, the handgrip exercise performed in the present study largely excluded the influence of body mass, and increases in total body mass may increase the relative intensity of locomotor based physical activity in individuals with MetS. For these reasons, this would be an interesting and valuable area of further investigation.

### Limitations

While interpreting the results of this study, there are a few experimental considerations that should be taken into account. First, the lack of MSNA recordings in this study does not allow for direct evaluations of sympathetic activity in individuals with MetS. However, it is well documented that both handgrip exercise exceeding 30% MVC and PECO elicit robust increases in MSNA (Cui et al., 2008; Delaney et al., 2010; Cui et al., 2016). For that reason, we remain confident that the blood pressure responses to the handgrip and PECO protocol used in the present study are strong indicators of overall sympathetic activity. Likewise, we do not report measures of peripheral blood flow, vascular conductance, or cardiac output, and therefore are unable to make explicit determinations regarding sympathetic vasoconstriction or central hemodynamic responses in the MetS group. Lastly, it should be noted that the sample of control subjects in the present study was not completely free of cardiometabolic risk factors. Specifically, four control subjects presented with two risk factors, and two others presented with one risk factor. Therefore, the observed differences between groups may actually be underestimated in this study. It is also important to note that the inclusion of individuals with one to two MetS risk factors is representative of the general US adult population.

### Future directions

There are a series of questions raised by the current study that are worthy of further investigation. First and foremost, it is important to elucidate the casual factors contributing to the development of exaggerated cardiovascular responses to exercise in individuals with MetS. As noted previously, neither resting blood pressure, FBG, nor WC were able to explain these adaptations, and the answer will likely be found by evaluating the neural and cellular adaptations that occur in parallel with disease progression. Understanding this will allow researchers and clinicians to begin working towards uncoupling these adaptations and preventing, or slowing, the development of cardiovascular dysfunction in MetS. Second, it would be valuable to understand the relative differences between contrasting MetS phenotypes (different groupings of cardiometabolic risk factors), and how these phenotypes influence autonomic dysfunction. It may be plausible that MetS, and the resulting impairments in autonomic control, develop differently based on which cardiometabolic risk factors present first, or are most severe. Investigators could examine this using a combination of MetS induced animal models and cross-sectional or longitudinal human studies evaluating different developmental stages of MetS. Likewise, it would also be important to understand how impairments in resting cardiac autonomic modulation, and particularly arterial baroreflex function, influences disease progression.

## Conclusion

The results from this study indicate that individuals with MetS demonstrate exaggerated exercise pressor and metaboreflex responses compared to control subjects, and that these differences occur independent of resting blood pressure, FBG, and central adiposity. This indicates that other MetS related risk factors likely contribute to the development of cardiovascular dysfunction in MetS, highlighting the complexity of this cardiometabolic condition. Future studies may consider identifying the specific risk factors, or groupings of risk factors, that contribute most heavily to the development of cardiovascular dysfunction in MetS, as this will identify the most appropriate targets for disease mitigation in this population.

## Data Availability

The raw data supporting the conclusion of this article will be made available by the authors, without undue reservation.
